# A Qualitative Study of Use of Mindfulness to Reduce Long-Term Use of Habit-Forming Prescription Drugs

**DOI:** 10.3389/fpsyt.2020.493349

**Published:** 2020-11-12

**Authors:** Ingrid Dundas, Kari Ravnanger, Per-Einar Binder, Signe Hjelen Stige

**Affiliations:** ^1^Department of Clinical Psychology, University of Bergen, Bergen, Norway; ^2^KOMPASSET, International Federation of the Blue Cross, Bergen, Norway

**Keywords:** mindfulness, qualitative study, prescription drugs, opioids, benzodiazepines, control, mindfulness-based relapse-prevention, thematic analysis

## Abstract

**Objective:** The aim of this study was to study how participants used a mindfulness-based program—Mindfulness Based Relapse Prevention to reduce use of habit-forming prescription drugs after long-term use. We explored participants' descriptions of their post-intervention strategies for controlling medication intake.

**Method:** Eighteen participants provided semi-structured qualitative interviews shortly after completion of the program and 13 participants were also interviewed 1 year after completion. Participants were asked about the conditions that originally led to use of medication, how they had attempted prior to the course to cope with problems associated with these conditions and their prescription drug-use, and whether and how their coping strategies had changed after participation in the program. Thematic analysis was performed within the framework of a realist epistemology, with an emphasis on researcher reflexivity.

**Results:** The following themes were identified in the material: Increased present-moment sensory awareness, Observing without controlling, Self-acceptance, Making conscious choices, Non-judgmental self-guidance, Sense of control, and Challenges of learning and using mindfulness.

Although these findings are closely related to the specific needs of our sample—mainly coping with tapering use of prescription drugs—they are largely in line with existing research on mindfulness interventions. An exception is the theme “Non-judgmental self-guidance,” which encompasses change in individuals' inner dialogue and practical self-care.

**Conclusions:** Analyses suggested that mindfulness might increase individuals' control over medication intake by shifting individuals' attention toward present-moment sensory awareness of concrete stimuli, thereby facilitating other kinds of control, such as non-judgmental inner self-guidance and more adaptive ways of reducing distress. We suggest that it is the moment-to-moment sensory awareness and non-controlling observation of distress, together with inner self-guidance, that differentiates mindful control from dysfunctional attempts at direct, top down control of medication-use.

## Introduction

Mindfulness-based interventions have been used for a range of clinical and non-clinical psychological problems, including general stress (1), depression and anxiety (2), pain (3), sleeping disturbances (4, 5), and substance abuse (6). Exactly how participants use the different aspects of mindfulness for coping with specific problems has attracted less research attention. This study aimed to explore the uses of mindfulness for achieving a specific purpose: reducing long-term use of habit-forming prescription drugs.

Benzodiazepine and z-drugs (benzodiazepine receptor agonists) are most often used for anxiety and insomnia, whereas opioids are most often prescribed for pain. Medical authorities in Norway, classify benzodiazepines, z-drugs and prescription opioids as habit-forming, and do not recommend them for long-term use (7). Studies have shown that continuous, long-term use may have adverse effects, such as cognitive decline in the case of benzodiazepines (8) and loss of analgesic effects and heightened pain sensitivity in the case of opioids (9, 10). Z-drugs were initially thought to be a safer alternative to benzodiazepines but have been found to be more prone to misuse and dependence than initially anticipated (11).

Qualitative studies have shown that both benzodiazepine users and prescription opioid users tend to be ambivalent about discontinuing their medication after long-term use and fear that reducing use may severely reduce their quality of life (12, 13). When deciding to reduce or discontinue use of these drugs, sometimes after several years of use, the user not only needs to cope with the distress that may have been the reason why the drug was prescribed in the first place, but with the additional self-regulatory problem of the urges, cravings, and fears about the consequences of not using the medication. Patients who are attempting to reduce or discontinue problematic, long-term use of prescription opioids are usually offered pharmacological treatments, but psychological treatments may be offered as an adjunctive therapy (14). The recommended treatment for patients reducing use of benzodiazepines is a stepped care approach, comprising letters of advice from the user's primary physician, short consultations, gradual, supervised withdrawal, and more specialist care and there is some evidence that adding psychological treatment to gradual dose-reduction regimes may increase the effectiveness of an intervention (15–17).

Many patients use both benzodiazepines and prescription opioids (18), so psychological interventions should ideally target all these drugs, so as not to reduce use of one at the cost of inadvertently increasing use of another. Some studies have indicated that mindfulness-based interventions may be useful for reducing use of z-drugs as well as prescription opioids. A case study by Lunde and Skjøtskift (19) reported that combining mindfulness meditation with cognitive behavior therapy had positive effects during tapering of medication for a woman with insomnia who had used a z-drug (eszopiclone) for 20 years. Another study (20) found that a mindfulness-based intervention—Mindfulness Oriented Recovery Enhancement (MORE) reduced harmful prescription opioid use.

Recent reviews of the effectiveness of mindfulness-based interventions have concluded that they are effective in treating addiction and addiction-related symptoms (21), especially when combined with TAU or other active treatments (22) and have a modest advantage over other clinical treatments, at least when patients complete the program of treatment (23). Mindfulness-based relapse prevention (MBRP) was developed to address challenges related to behavioral control in the context of drug and alcohol cravings. Mindfulness-based relapse prevention is an 8-week, group-based program that has been shown to help prevent relapse to substance abuse [e.g., (6, 24, 25)]. As with most mindfulness interventions, the techniques involve attending to moment-to-moment inner experiences such as the body or breathing and bringing attention back to this “anchor” whenever it wanders. This moment-to-moment observing should be done non-judgmentally, meaning without trying to change or judge what is happening at that moment. This may counter tendencies to act on autopilot, i.e., act automatically, out of habit. The “SOBER” exercise is central to MBRP. It involves Stopping, Observing one's inner experience and outer context in the present moment, attending to one's Breathing, Expanding awareness to the body as a whole and one's current situation, and Responding in ways that are consistent with one's values and goals. Research suggests that MBRP may strengthen people's ability to monitor and cope with discomfort associated with craving for substances such as alcohol and illicit drugs (25). A recent meta-analysis of randomized controlled trials, reported that MBRP, although not better at preventing relapse than other interventions, had a greater effect on withdrawal, cravings, and negative consequences of substance abuse (26).

To our knowledge there have been no qualitative studies of users' experiences with MBRP as a technique for helping people to reduce or discontinue long-term use of habit-forming prescription drugs. This study explored the first-person accounts of individuals who wanted to reduce or discontinue long-term prescription drug use and had signed up for a MBRP course in order to find out whether and how mindfulness might help them to achieve this goal.

## Materials and Methods

### Procedure

Courses were led by clinical psychologists trained in MBRP who also had prior experience of working with substance abuse disorders (the first course was led by the first and second author, the remaining three courses by the second author). Courses adhered to the MBRP manual (24) and consisted of eight 2.5 h sessions. After completing their course participants were interviewed at the local university by one of three psychologists none of whom had been involved in delivering the courses. Interviews took ~1 h. Participants received a 200 kr (25 US dollars) gift card after their last interview.

Participants were interviewed shortly after their course, and again 1 year on from their course, about the conditions that had led to their use of medication, how they had previously attempted to cope with problems associated with these conditions and whether their coping strategies had changed since their participation in the program. The interviews were conducted according to Kvale and Brinkmann's (27) guidelines for checking and validating the interviewer's interpretations during the interviews. Descriptions of concrete episodes were elicited whenever possible, in order to increase the chances that the interviewer and interviewee were speaking about the same thing (28). Interviews were not conducted by the facilitator of the course that the interviewee had attended.

### Participants

Our target group was individuals who had originally been prescribed medication classified as habit-forming. We chose to interview individuals who had used one or more of the targeted drugs for at least 1 year. Four MBRP courses were held over a period of 2.5 years in order to collect enough interviews to reach saturation. Participants were recruited via newspaper advertisements and information leaflets distributed by their physician or psychologist and were screened during an intake interview before participating in the courses. Inclusion criteria were long-term (>1 year) use of habit-forming prescription drugs, a desire to reduce or discontinue use, and having physicians who recommended discontinuation. Exclusion criteria were having conditions needing more intensive or different treatments, i.e., suicidal plans or psychotic episodes in the previous year; ongoing, extensive use of illicit drugs; untreated alcohol problems; serious anorexia. Two potential participants were excluded at intake because they did not wish to reduce their medication. Two participants with pain whose medication was not listed as habit-forming were allowed to attend the courses, but their data were removed before analysis in order to limit the sample to individuals on long-term habit-forming medication. No other participants were excluded.

Of the 25 participants who enrolled in the courses, 18 completed their course (14 women and 4 men, mean age 52 years, *SD* = 7.9 years, range 35–67 years). Challenges starting their prescription drugs use included chronic pain, sleeping problems, anxiety, and depression. Frequent doctor visits and contact with psychologists or physiotherapists was common. Approximately two thirds of the participants were working part- or full-time, but the study also included pensioners, participants on long-term sick leave and participants on disability benefits. All were Caucasian, and all but one was born in Norway. Their education ranged from a university level degree to basic schooling.

The completers were interviewed individually shortly after their course (*n* = 18), and 1 year later (*n* = 13). In addition to several drugs that were not classified as habit-forming, 16 of the 18 completers used z-drugs and/or benzodiazepines; four were using them combination with prescription opioids. Two used prescription opioids in addition to non-habit-forming drugs, but not benzodiazepines or z-drugs. All participants reported having used their habit-forming drugs for extensive periods of time (*M* = 12 years, range = 1–27.5 years). We used a slightly modified version of the Benzodiazepine Dependence Self-Report Questionnaire [Bendep—SQR; (29)] to examine self-reported dependency at baseline. The scale was modified to capture dependency on all three classes of habit-forming drugs. The Bendep-SQR has shown acceptable psychometric qualities (30, 31) and showed good reliability in our sample (Cronbach's alpha = 0.72). Seventeen of the 18 completers had “high” or “very high” cores for problematic use and one had a “moderate” score.

### Analytical Approach

Interviews were transcribed verbatim and subjected to inductive semantic thematic analysis (32). Thematic analysis is a flexible approach that allows the researcher to identify and describe patterns (themes) in a qualitative dataset and to develop hypotheses based on the data. Our epistemological stance lies somewhat toward the realist end on the constructionist-realist epistemological spectrum, although we agree that researchers influence data in numerous ways, e.g., when deciding upon research questions, conducting interviews, and interpreting and writing up findings. Although we strove to ensure that the themes that emerged from the analysis reflected the voiced experiences of the participants, the hypotheses about relevant processes (described and discussed in the Discussion part of this article) were constructed by us on the basis of our findings and the literature.

As recommended by Miles and Huberman (33), the analysis progressed from a bottom-up coding phase to intermittent, concept-driven coding as themes started to crystallize. We started by familiarizing ourselves with the transcripts [phase 1 in (32) steps for conducting thematic analysis]. The first author then carried out a bottom-up coding of the transcripts, focusing on participants' perceptions of the usefulness of mindfulness in their efforts to reduce use of habit-forming prescription drugs, particularly any reports of changes after the course or problems in using mindfulness. She used the Nvivo program to generate codes as she read, trying to put aside her pre-conceived ideas about what she might find (Phase 2 of Braun and Clarke's procedure).

Braun and Clarke (32) recommend that writing should be an integral part of analysis and begin in phase 1. The first author shared and discussed written accounts of relevant categories and themes, together with relevant citations, with the other authors. After each discussion she revisited the transcripts and relevant literature again, sometimes changing how the material was organized. After several iterations the final coding structure was agreed upon by all the authors and the report written (phases 3-5 of Braun and Clarke's procedure).

Where excerpts from interview are quoted in the Results section, information from other parts of the interview is presented in square brackets. The term “most of the participants” refers to more than half, “some” refers to five to nine participants, and “a few” refers to <5 participants. T2 signifies that the excerpt is from an interview carried out 1 year after the interviewee had completed the MBRP course; all other excerpts are from interviews carried out shortly after the interviewee had completed the course.

## Results

During the interviews all but one participant described previous attempts to reduce use of the target drugs, either with the help of professionals (psychologists, physicians, and physiotherapists) or on their own. One had been hospitalized for complications related to excessive use of medication. Reasons for wanting to reduce or discontinue use were: unease about feeling dependent (often after unsuccessful attempts to reduce or discontinue use); diminished effectiveness of the medication; being advised to reduce or discontinue use by a physician; unwanted cognitive or affective effects (e.g., feeling dull, memory problems, and/or flat affect). Analyses of perceived changes after the course yielded six themes, described below. We also describe the challenges participants encountered whilst they were learning and using mindfulness.

### Increased Present-Moment Sensory Awareness: Noticing All the Things One Usually Takes for Granted

Participants often referred to increased moment-to-moment sensory awareness when asked if anything was different after the course. One participant responded:

A lot is different. Very different. My use of medication has fallen drastically. I've become more observing of the good things [in life], the things that make me happy… I've gone for walks in the woods… seen birds. In a way, I've noticed all the things one usually takes for granted. (Participant B).

The same woman spoke about how the way she related to her young son had changed: “I'm able to sit down with him and enjoy the moments in quite a new way… I'm more present in my life.” This awareness seemed to involve an awareness of moment to moment concrete sensory input. A woman with chronic pain (Participant A) said that after the course she was more aware of “the creaking in the snow, the sound of water in the creek, sounds of birds, and the color of the leaves on the ground.”

This type of moment-to-moment awareness was sometimes contrasted with being pre-occupied with abstract thoughts. A participant explained, “I get happiness from that which is present in the moment. Yes, that's it. Instead of working in my head on something that might happen later.” (Participant C, T2).

### Observing Without Controlling: Managing to “Uncouple” Oneself From Distressing Thoughts

Most participants said that after the course they were able to observe their feelings and thoughts without attempting to influence or control them, often using spatial terms to describe their experiences, e.g., “letting go,” “letting things drift by,” and “disengaging.” One participant explained, “the course has given me a kind of distance, I can stand on the outside [and observe] and then kind of [choose to] take it [a distressing thought] to heart or not.” (Participant D). Another woman explained:

I breathe in a way that makes me calm inside. That gives me some distance from … [the distress], or you might say that I get some relaxation or rest. I manage to “uncouple” myself from it. (Participant E, T2).

Abstaining from attempts to control thoughts and feelings was contrasted with rumination. One participant noted that, prior to the course, painful thoughts about difficult experiences would tend to “grind around and around, like a grinding stone” and contrasted this with his increased ability to let such thoughts occur, without elaborating on them, since the course:

[I've developed] a more receptive mind and greater tolerance for events that used to hurt me and used to draw me into a state of rumination… Instead, I now have a more relaxed relationship with these thoughts. (Participant F).

### Self-Acceptance: No Longer Hitting Oneself Over the Head

Observing thoughts and feelings without trying to control them seemed to reduce the associated self-blame and upset. For example, a woman explained that she no longer sought to reduce distressing thoughts and feelings as soon as they occurred: “[instead of thinking] ‘oh, it's *so unwise* to be so stressed,’ I just let the stressful feelings continue” (Participant E). Another participant noted that he “let go” and moved on, rather than worrying about things that had happened and his own shortcomings:

I accept more of myself now, or accept myself more than I used to, I believe I trust myself more. Before I was very judging of myself, didn't like myself. [I was] worried and bothered by things that had happened, things that came back to me all the time and gnawed at… (draws breath). But now I am more accepting of myself, both my strengths and weaknesses… I accept my weaknesses, too. I no longer hit myself over the head afterwards, telling myself how stupid I've been… I let go and go on. (Participant F, T2).

### Making Conscious Choices: Reflecting Before Taking a Pill, and Sometimes Not Taking It

Making conscious choices was contrasted with automatic responses. A participant with chronic pain described the process:

Those moments when one actually makes a conscious choice: shall one take something, does one need medication at the moment? Can I wait a little? Can I take less? How is my body, really? Are there other [work-related] tasks I might start working on instead? …You turn [your chair] around, you stand up instead of remaining seated. You adjust the challenges that you face. (Participant G).

Participant B, who had been using benzodiazepines for 7 years and prescription opioids for 4 years, said that the course had helped her “consider a little, before opening the medicine cupboard.” She said “Before I would just walk right over [thinking] ‘if I need them, I need them.’ I took them together with my other medication [as a part of my routine]” (Participant B).

A man troubled by social anxiety explained that since the course he would consciously reflect on his choices before using medication. He contrasted this to his former habit of using medication before attending threatening social events: “[I] reflect a bit, before I take the pill… in contrast to automatically [using medication]… the way I used to before entering a social setting. [Now I] maybe… postpone it a bit more often than I used to.” (Participant H).

### Non-judgmental Self-Guidance: What Else Might You Do?

As part of the process of becoming aware of their choices, some participants described guiding themselves in ways that helped them respond differently. For example, participant E explained:

Now I know that if I need to enter a setting that I used to need medication to enter, I can instead talk to myself and say, ‘You know, it's actually all right that you might feel sad when in that situation, because it's human, it's totally okay, that [feeling].’ So that is, you know, a totally new way of thinking. (Participant E, T2).

A woman with chronic pain explained that prior to the course she would tell herself she needed medication immediately when distressed. Since the course this had changed:

When the question pops up ‘should I take another pill, should I?’ – at that moment [I might say to myself] ‘only two hours until I can go home’. You see, not telling myself ‘you can't have a pill’, but instead ‘not just yet’. You cope a little bit and then a little bit more [and tell yourself] ‘breathe and don't get all stressed out’… Talking to myself like that [helps]. (Participant G, T2).

Participant F explained that when he found himself ruminating, he would tell himself to just “let them [the ruminations] sail by. Don't analyze them now.” Often participants' self-directed inner guidance resembled the guidance provided during meditations. Participant G offered an example of being caught in a hectic situation in which she was in pain and impatient but had to stay standing up. She explained that she allowed the pain to be present and did a standing body scan while “talking to” her different body parts:

Well, the other day I was standing in a queue. And time was running out and I really didn't have the time to stand there. So I started talking to my knees… Since I had no other choice but to just relax, and be present where I was, you see. From my knees to my hips to my back… and let my shoulders drop… That kind of talking to oneself… ‘Yes, I'm *me*, and I am contributing in the way that is possible for me to contribute, here at this moment, and my body is present and I'm breathing calmly now.’ You see, it's a way of speaking to yourself that can help in an otherwise hectic situation. (Participant G, T2).

### A Sense of Control: There Is Something I Can Do

The final theme involved having a sense of control and a repertoire of manageable ways of working with pain and distress, and was described by most participants. This was often related to a greater awareness or understanding of oneself and one's situation. For example, one woman described having a type of control that was based on an awareness of her habits “The difference [since the course] is that I've become very aware of my thoughts, and patterns. And now when I have this awareness, I feel that I have control.” (Participant E). She contrasted this to her former sense of helplessness in the face of strong feelings and habit of taking her medication “just in case.”

Another woman described feeling in greater control of her responses to physical pain, having realized she had the ability to influence her situation and the related pain:

[The course has helped me to] be consciously aware of the fact that it is you, yourself, who chooses what to do next… I value [having gained] the experience of having a way of influencing [my situation and pain]… that there is something I can do…. [I have learned] that I can influence it, I can choose… just to observe, and then do something about it [such as rising from a sitting position, moving, or carrying fewer items when shopping]. (Participant G, T2).

One woman (who had managed to discontinue benzodiazepines after 5 years of use and was still refraining from use 1 year after the course) explained that she had learned to slow down and stop and that helped her to prevent her thoughts and feelings from running out of control. Noticing that she was able to use the mindfulness techniques she had learnt to calm herself made her feel more in control:

I use that ‘stop’ practice daily, that stopping in your mind, stopping and managing to stay with it… slowing down and putting on my brakes just a little, so that I'm not – what shall I say – run away with, but able to stop myself… even though it's difficult, I believe it's possible to practice, so that you become calmer in your body, so that you have a greater control. I've become much more aware of being able to calm myself. (Participant I).

In addition to the more informal uses of mindfulness described above, taking time to practice formal sitting meditation was mentioned by one participant as being helpful in controlling the urge to use medication: “Some days, when I felt I needed my medication…, I have meditated. This enabled me to avoid taking a pill. It has helped me through… even during quite stressing situations.” (Participant F).

### Challenges With Learning and Using Mindfulness

Although MBRP was mostly described in positive terms, participants also mentioned problems with learning and using mindfulness. Greater moment-to-moment sensory awareness could be difficult when awareness was drawn toward uncomfortable thoughts and feelings. Participant I noted that during the course she sometimes felt conflicted about the suggestion to be aware, preferring just to “flow through the day” without too much awareness. Three of the 18 participants described how trying to observe their thoughts and feelings without controlling them actually increased their pre-occupation with them, evoking inner protests against accepting pain, fears that thoughts might become real and an acute need for distraction from those emerging thoughts and feelings. One participant explained:

[I have had] so very many thoughts, and disquieting thoughts (…) When things are quiet [during meditation], these thoughts sort of emerge even more” (Participant J).

The consequences of not using habit-forming prescription drugs were also a source of worry. Participant K, who described needing to control her drinking due to alcoholism as well as suffering from insomnia, compared the consequences of abstaining from excessive alcohol use and abstaining from sleeping medication. She felt that abstaining from medication was more difficult and had more serious consequences than abstaining from alcohol, because she would be unable to cope with her day and also less likely to abstain from alcohol if she was fatigued. A few participants said that during the course they had decided to make a smaller reduction in their medication use than they had previously planned, because they felt they needed it in order to retain their working capacity and function in society. Other problems included difficulty concentrating during the sessions, restlessness, impatience, tiredness, and extreme bodily laxity during formal meditation.

## Discussion

In general, the themes that emerged from our analysis are in line with previous studies of mindfulness interventions for other populations. The first theme, increased present-moment sensory awareness, has been described as opening up to and appreciating pleasant experiences, as well as enhanced presence when relating to others across a wide range of samples (34). Garland et al. (35) cite evidence that training in attending selectively to natural rewards (such as nature, and positive relationships with close others) rather than drug rewards may be one important pathway through which mindfulness may work to reduce dependence on opioid prescription drugs. They suggest that mindfulness-training will “strengthen top-down cognitive control to restructure bottom-up reward learning from valuation of drug reward to valuation of natural reward” (p. 3).

The second theme, observing without controlling, was described by Malpass et al. (36) in their meta-ethnography of qualitative studies on mindfulness-based interventions as a process of “stepping back” and “letting go.” The third theme, acceptance, has also been a frequent theme in qualitative studies of mindfulness (36). The fourth and the sixth theme, awareness of choice and the sense of control, have both been noted in several other studies of mindfulness-based interventions (36–41).

Garland et al. (20) reported that their mindfulness-based intervention for discontinuing use of prescription opioids reduced the severity of participants' pain and the extent to which it interfered with their lives, and that these effects were mediated by reinterpretation of pain sensations as innocuous sensory experiences such as numbness and by increased “non-reactivity” as operationalized by the Five Facet Mindfulness questionnaire. Our participants described attending to the breath while “letting things drift by,” “disengaging” and “uncoupling.” We chose to call these responses “observing without controlling,” but they might also be termed “non-reactivity.” Non-reactivity as measured by the FFMQ refers to a capacity to observe without impulsivity, to “just notice and let go” (42).

Another study of the mechanisms involved in reducing substance abuse through mindfulness suggested that the reductions in substance abuse during a MBRP might be due to a reduction in craving, which was attributed to increased awareness with acceptance and non-judgment (43). We suggest that the responses that we have labeled “observing without controlling” could also be described as awareness with acceptance and non-judgment.

Our fifth theme, which we have labeled “kind self-guidance,” is mentioned less frequently in the mindfulness-literature. In times of distress participants would stop and attend to the present moment and speak kindly to themselves. This inner speech could be calming (e.g., “this is just a feeling, that's what it is”), a reminder that some situations did not call for immediate action (e.g., “You can't do anything about this at the moment anyhow”) or a suggestion about what to do next. It was non-judgmental and often practical. Although self-care is a component of the MBRP program, this conscious and friendly inner self-guidance was not explicitly taught as a part of the intervention but seemed to emerge naturally. It is possible that training in mindfulness increases autonomous use of non-judgmental inner guidance resulting from such guidance during training. Such inner talk might influence feelings in the same way that loving kindness meditations contribute to “cultivation of wholesome emotions” [(44), p. 154].

Although participants generally spoke about the course in positive, often enthusiastic, terms, they also described problems, such as difficult feelings when “just observing” inner experiences. There is wisdom in encouraging participants to “welcome” into awareness without judgment even very difficult feelings. For example, MBRP's “urge surfing” exemplifies how moment-to-moment experience of craving can be welcomed (or at least tolerated), until they pass. The urge is observed closely from moment to moment, but not acted upon. However, emotional pain and confusion may be so overwhelming as to be retraumatizing for some individuals (45). In such instances, the ability to non-judgingly observe one's emotional pain is temporarily lost, and the emotions are no longer seen as impermanent events in the mind. In such instances, participants may need support from the facilitator and help to reorient themselves. Also, for some illnesses, e.g., rheumatic arthritis, facilitators may need to remind participants that movement is permissible, since physical pain from immobility may be a healthy reminder of the need for movement to prevent increased joint-inflammation. The increasing pain is accepted as a signal, not just passively observed. This illustrates the limits, in some circumstances, of accepting without acting.

### Some Hypotheses About Processes at Work: Increasing Control by Releasing Control

One strength of qualitative studies is that they can provide the foundations for development of hypotheses and theories. It may seem paradoxical that mindfulness training, with its emphasis on non-control of inner experiences, can be useful to people who are trying to exercise greater control over emotional and physical distress. In the following we suggest some processes that may be involved.

Participants described stopping and attending closely to concrete sensory experiences, such as moment to moment sensations of the breath and body, as well as outer sensory experiences in nature, such as sounds of birds. Moreover, participants described valuing these stimuli more. Valuing natural rewards is a capacity that is explicitly taught in other mindfulness based interventions such as MORE and that may be essential when treating addictions (35).

Participants also described a sense of releasing control. Words they used were letting go, uncoupling, letting things drift by, and disengaging. We suggest that this letting go was facilitated by closely attending to concrete inner or outer sensory experiences. As suggested by Dorjee (44) training people to attend non-elaboratively to their inner experiences can bring about a shift of attention from “higher-level” processing, including language and thought, to “lower-level” sensory input (e.g., moment-to-moment sounds in the environment or somatosensory perceptions). Hölzel et al. (46) argue that although mindfulness might initially require some top down control (e.g., when deciding to “mindfully” direct one's attention toward one's breath or other sensory input), mindfulness might also involve reduced cognitive control.

Participants also described experiencing a sense of distance or disengagement. Words used were gaining distance, standing on the outside and observing. This was associated with the stopping and attending to concrete sensory input from the body. It may seem strange that closely attending to sensory input from the body should be experienced as providing a distance to distress, since it would be natural to expect that attending closely to the body would increase awareness of distress. However, distress consists of one's interpretations of bodily sensations and pre-occupations of what these situations may mean in addition to the “raw” sensory input. It has been suggested that attending to concrete sensory impressions may help someone “disengage from pre-occupations with which she may otherwise be attending” [(47), p. 296]. Often distress will cause people to become more pre-occupied with their own faults, problems and worries, and aspects of their experience that are not as they feel they should be. On the basis of the descriptions provided by our participants, we suggest that attending to immediate sensory input enabled the individual to “stop” and “let go of” maladaptive pre-occupations (such as the urge to end the distress with medication and pre-occupations with thoughts about the meaning of the sensations), at least for a while. We suggest that directing non-judging attention toward concrete moment-to-moment experience might have helped participants disengage, at least momentary, from dysfunctional pre-occupations.

But why did attending to sensory input and disengaging from dysfunctional pre-occupations provide a sense of greater control? Our participants noted that they would disengage and not be as “run away with” or swept up by feelings. It seems that directing attention to concrete sensory stimuli (the breath) combined with decreased top down cognitive control, may be one way of down-regulating emotional (48) as well as physical (49, 50) distress. Farb et al. (48) argued that the capacity to regulate emotions “mindfully,” differs from traditional “top-down” cognitive efforts to control negative emotions by two processes: “attention to present moment sensation” and the “suspension of judging experience to be intrinsically good or bad” (p. 71). In a similar vein, Leary et al. (51) proposed that there exists a form of self-regulation (hypo-egotic control) that requires the individual to refrain from the usual conscious attempts at self-control and is facilitated by attending to concrete, moment-to-moment sensory stimuli. It seems that attending directly to present moment stimuli as simply sensory stimuli (lower-level processing) rather than thinking about them (higher-level processing) might facilitate emotional regulation and control of reactions to distress, perhaps via a suspension of judging experiences to be good or bad and a releasing of pre-occupation with own faults or with the need to immediately change distress via medication.

The present participants also described engaging in inner verbal self-soothing guidance of how to proceed. This seemed to contribute to their ability to post-pone outer action and to make a conscious choice about whether to proceed by using medication or not. We suggest that engaging in such verbal self-soothing directed attentions away from unhelpful inner pre-occupations and toward more optimistic possibilities.

Increasing control by releasing control may seem incompatible with the view that self-control involves the act of overriding impulses. Baumeister et al. (52) defined self-regulation as the ability to override one's cognitive, affective, and action tendencies in order to behave consistently with one's values and goals—even when feeling a strong wish to do something else. One would expect individuals with a long history of relying on medication to need to pull themselves together and override their cognitive, affective, and action tendencies in order to succeed. However, we suggest that an ability to override impulses is not incompatible to letting go of cognitive control of inner experiences. Our participants described refraining from exerting their automatic responses (taking medication) by directing attention to concrete stimuli on the one hand, and “letting go” and “distancing” on the other hand. The overriding of automatic responses after mindfulness training has been attributed to both top-down and bottom-up processes [e.g., (43, 53)]. Bottom-up processing may be defined as processing sensory information as it is coming in. We suggest that the overriding of habitual drug-taking responses that our participants reported was facilitated by the experience of calm and non-reactivity inherent in attending to moment-to-moment concrete sensory inner experiences (e.g., one's breath) that is: “observing without controlling.”

This “observing without controlling” may also be applied to thoughts and feelings, involving the attending to moment-to-moment aspects of these inner experiences “as from a distance,” that is, without undue cognitive elaboration. As mentioned, this way of processing thoughts and feelings make these thoughts and feelings less upsetting and thus may function as a way of regulating emotions. Participants expressed satisfaction with having learned they could “just observe” thoughts and feelings rather than being “run away with” and described feeling generally more aware after the course and being able to refrain from automatic, habitual responses. The moment-to-moment sensory awareness and observation without control differentiates mindful control from attempts at dysfunctional control via thought suppression, preservative cognition (such as rumination and self-blame), and problematic uses of medication.

Garland et al. (54) and Garland et al. (35) cite evidence that mindfulness-training may contribute to reduced harmful drug use via strengthening top-down cognitive control of attention, set-shifting capacity and an increased ability to savor healthy stimuli. The experiences of our participants' support this. Our participants reported stopping their automatic action tendencies by consciously directing their attention to the concrete, moment to moment aspects of their inner sensations (top down cognitive control and set-shifting). Participants also reported finding increased pleasure in noticing the sensory aspects of nature and interpersonal interactions (savoring).

The possible role of insight in explaining the experiences of the participants in this study is worthy of acknowledgment. In Buddhism, diligent practicing of “right” mindfulness brings about understanding of the three marks of existence: the ubiquity of suffering (suffering is a part of living), the fact that experience and existence are constantly changing (impermanence) and the actual impersonal and interdependent nature of experience and phenomena (noself) (55). Such realizations may increase a sense of control. For example, an awareness of the impermanence of their current distress was implicated when participants chose to wait before taking a pill. Several participants said that they gained greater awareness of their thoughts and “patterns” during the course, and “looked at things in a different light.” When asked to give examples, they described becoming aware that thoughts were just thoughts and could be let go of rather than acted upon, that attending to the breath would increase one's ability to function in anger-provoking interpersonal situations, that one could make a conscious choice of whether or not to take a pill at a particular moment, and that one could be aware of colors and sounds in nature. Some also mentioned realizing that they had difficult feelings inside that they had avoided thinking about. They described many of these realizations as discoveries that increased their conscious awareness of what they might do, and that this awareness sometimes gave them greater control. Further study might explore whether and how a greater understanding of the three marks of existence might develop after participating in MBRP.

Unique for this study, is a description of how participants spontaneously developed a form of self-guidance. Participants became their own instructors and directed their own attention and behavior. Further studies of such self-guidance could be clinically useful. Does this tendency to guide themselves, generalize to other samples? How do different people guide themselves when they attempt to reduce unhelpful drug use? According to Buddhist thinking, certain objects of attention can be “unwholesome.” As noted by Anālayo (56) “an object [of concentration] could in principle also be an unwholesome one. If something triggers anger, for example, this could have quite devastating effects if the mind has previously been trained in the ability to stay focused but not in the ability to discern clearly what is unwholesome (viz. right view) and to monitor closely what is taking place (viz. right mindfulness).” We suggest that studying whether, and under which conditions, people develop wholesome self-guidance, could be useful. Also, examining if individuals who spontaneously use certain types of self-guidance are more successful in achieving their outcomes, would be interesting. Since spontaneous self-guidance might not be immediately accessible in awareness, one might want to ask participants to observe what they “say to themselves when using mindfulness” after a MBRP course. If one wanted to trigger inner self-guidance in experiments, one could administer two mindfulness meditation scripts: one containing suggestions that participants might want to guide themselves when trying to abstain from using a substance at anyone specific time, the other identical but without the suggestions for self-guidance. Our hypotheses would be that helpful self-guidance would be characterized by direct, concrete, moment to moment attention (e.g., to the breath) coupled with the sense of observing without controlling as described in the present study, and any additional helpful comments, such as “just 2 h, and you can go home” or “you're standing here, and that's ok, just feel your legs.” We would expect that less helpful responses would include judging what has been or might become, as in worry or rumination (however, reminding oneself of the drawbacks of use might be useful). Attending to a drug reward without the observing distance needed to discern this as an unwanted/unwholesome focus would probably be unhelpful, as has been suggested by Garland et al. (35).

Summarizing, this study adds to the literature by emphasizing the role of intentional non-control and inner guidance when trying to reduce prescription-drug use. Without the option of focusing attention on concrete moment-to-moment sensations and letting go of attempts at control, attention may easily be usurped in attempts to exercise top-down conscious control (including willpower) to try to find a way out. When solutions are not available (since neither use nor non-use seems acceptable at that moment) futile attempts to control distress and urges for relief may exhaust the “control muscle” (52). We have suggested that mindfully observing concrete moment-to-moment inner experiences while not trying (at that moment) to find a solution but instead letting go of attempts to control distress, together with inner kind self-guidance (e.g., “you're doing fine, attend to your breath”), could reduce the frequency of engaging in unwanted prescription-drug use.

### Limitations of the Study

To our knowledge this is the first study to describe the experiences of participants who attempt to reduce or discontinue long-term use of prescription drugs with the help of mindfulness. Our participants were probably less familiar with Western mindfulness jargon than younger people or Americans and seemed to have few ready-made explanations of how mindfulness might feel and work. The descriptions of experiences we collected were detailed, concrete, and mostly consistent within and across participants, which increases our confidence in the validity of the results.

Our sample consisted a small sample of self-selected and fairly strongly motivated participants, mostly middle-aged women, who had been prescribed the drugs on which they had become dependent. This may mean that our findings do not generalize to, for example, people using the same types of drug for recreation and/or illegally or to young men. Larger studies with participants with more diverse cultural backgrounds are also needed. Second, the ways in which our sample used mindfulness are probably not the only ways of using mindfulness to manage one's intake of prescription drugs. Nor do we believe that the processes we suggest on the basis of their descriptions are the only processes involved, there are probably several other processes that are also involved. Third, combining participants using different kinds of drugs into a single sample may have masked drug differences in the ways in which mindfulness can be of use. Fourth, the design of this study did not allow us to investigate whether changes in underlying pathology (e.g., anxiety, insomnia, or depression) mediated perceived ability to control medication intake, as has been suggested by Gould et al. (15). Finally, this study was not designed to evaluate how successful participants were in reducing their medications.

It is worth reflecting on our roles in this study, as researchers and facilitators of the MBRP courses. Two of us have been trained in MBRP, which may have directed our attention toward the usefulness of this approach. Although participants spoke freely of times when it was not feasible for them to abstain from using medication, they may have been inclined to emphasize positive change due to demand characteristics of non-blinded intervention studies.

## Conclusions

Analyses of the interviews suggest that mindfulness can help individuals release habitual attempts of controlling inner experiences via medication or otherwise, and to find alternative forms of control. These alternative forms of control seemed to involve shifting attention to moment-to-moment concrete sensory input and friendly inner guidance. This differentiates mindful control from dysfunctional attempts at controlling inner experiences via thought suppression, rumination, self-blaming, and unwanted uses of medication.

## Data Availability Statement

The datasets generated for this study will not be made publicly available. The data consist of interviews and we have not collected permission from the participants to make the datasets available.

## Ethics Statement

The studies involving human participants were reviewed and approved by The National Review Board Regional Committee for Medical and Health Research Ethics (2009/1294). The patients/participants provided their written informed consent to participate in this study.

## Author Contributions

ID and KR conducted the MBRP courses. All authors took part in preparing, finishing this manuscript, and agree to be accountable for the content of the work.

## Conflict of Interest

The authors declare that the research was conducted in the absence of any commercial or financial relationships that could be construed as a potential conflict of interest.
